# Reply to Asgarian et al. Comment on “Nejati et al. Spatiotemporal Patterns and Historical Overview of *Aedes* Mosquitoes in Iran: A Systematic Review. *Trop. Med. Infect. Dis.* 2026, *11*, 131”

**DOI:** 10.3390/tropicalmed11090239

**Published:** 2026-08-24

**Authors:** Jalil Nejati, Abedin Saghafipour, Rubén Bueno-Marí

**Affiliations:** 1 Health Promotion Research Center, Zahedan University of Medical Sciences, Zahedan 98167-43463, Iran; jalilnejati@yahoo.com; 2 Department of Public Health, Faculty of Health, Qom University of Medical Sciences, Qom 37169-87366, Iran; 3 European Center of Excellence for Vector Control, Laboratorios Lokímica—Rentokil Initial, 46980 Valencia, Spain; ruben.bueno@uv.es; 4 Parasites & Health Group, Department of Pharmacy, Pharmaceutical Technology and Parasitology, Faculty of Pharmacy, University of Valencia, 46100 Valencia, Spain

We thank Asgarian et al. for their careful reading of our article and for highlighting points that merit clarification [1]. Our review was intended as a systematic synthesis of eligible published literature on *Aedes* mosquitoes in Iran based on predefined criteria, rather than as an exhaustive inventory of all the historical records, governmental surveillance documents, and gray literature [2]. We nevertheless acknowledge that some of the issues raised, particularly those related to internal consistency between the text, tables, and map, as well as the completeness of species listing, are important, and these are addressed in the following response.

We agree that the discrepancy between the total number of *Aedes* species stated in the text and those listed in Table 1 of [2] represents an inadvertent omission that requires clarification. We thank the authors of the Commentary for drawing attention to the absence of *Aedes asiaticus* from the table. This omission should be considered in the context of the taxonomic history of the species and the temporal scope of our review. *Aedes asiaticus* was historically treated as a subspecies of *Ae. pulcritarsis* and has only recently been recognized as a separate valid species following the taxonomic reassessment by Harbach and Wilkerson (2023) [3]. Consequently, many earlier Iranian records did not distinguish between the two taxa. Moreover, as noted by Azari-Hamidian et al. (2025), although both *Ae. asiaticus* and *Ae. pulcritarsis* are currently included in the Iranian mosquito fauna, no precise information is available on the distribution of *Ae. asiaticus* in Iran [4]. Historical reports (e.g., Monchadskii, 1951) often provided inexact localities and predated our January 1980–October 2025 eligibility period [5]. Studies published within our review period primarily reported *Ae. pulcritarsis* and did not provide distinct locality records that could be attributed with confidence to *Ae. asiaticus*. These circumstances explain why no separate spatiotemporal distribution record for *Ae. asiaticus* was included in our synthesized dataset; however, they do not justify its omission from the taxonomic list. We therefore acknowledge that *Ae. asiaticus* should have been listed in Table 1 of [2] as the fifteenth species for taxonomic completeness. The present response clarifies this omission and the distinction between taxonomic recognition of the species and the availability of eligible species-specific distribution records within the temporal scope of our review.

We appreciate the commentator’s concern regarding the potential omission of governmental and technical reports, including materials disseminated by the Iran Center for Disease Control (ICDC). We fully acknowledge that the ICDC has reported the presence of *Ae. aegypti* in Fars Province and *Ae. albopictus* in Zanjan and Qazvin Provinces. However, our review was restricted to published studies with accessible full texts in Persian or English, and therefore did not capture governmental or technical reports available through institutional websites or other gray-literature sources. Following the commentator’s suggestion, we additionally re-examined the peer-reviewed and the published literature to determine whether these province-specific records had been independently corroborated; we did not identify eligible studies confirming these specific records within the scope of our search. We agree that incorporating the gray literature may improve future updates to the evidence base. However, the absence of a province from our distribution map should be interpreted as the absence of eligible records in the reviewed dataset, not necessarily the biological absence of Aedes in that province.

We thank the authors of the Commentary for pointing out the omissions of Keshavarzi et al. (2017) and Kayedi et al. (2020). We confirm that these studies were inadvertently omitted from our review despite being relevant to the evidence base. Specifically, the study by Keshavarzi et al. (2017) reports *Ae. vexans* in Fars Province and the study by Kayedi et al. (2020) reports *Ae. vexans* for the first time in Lorestan Province [6,7]. As a result, Fars and Lorestan Provinces were incorrectly coded as “Not reported” in Figure 2 of [2]. We acknowledge these omissions. To clarify the record, both provinces should be recognized as having documented records of *Ae. vexans* based on these findings. We would like to clarify that our systematic review process was not limited to abstracts; full-text screening was a mandatory step. These omissions represent unintentional clerical oversights during the final data extraction and mapping phase rather than a systematic failure to review full texts.

In contrast, the record of *Ae. pulcritarsis* from Semnan Province reported by Gutsevich (1943) [8] falls outside the temporal scope of our review and was therefore not eligible for inclusion. Although this record was cited again by Azari-Hamidian and Harbach (2025) [4], this does not change the original date of the occurrence record. Therefore, the “Not reported” label in Figure 2 of [2] refers to the absence of an eligible record within the review period, not to the absence of any historical record from the province.

We would like to clarify that the statement in Section 3.2.2 of [2] regarding Kurdistan Province, where we noted that “mountainous and cooler regions such as Kurdistan and Lorestan yielded no *Aedes* specimens in several surveys”, refers specifically to the results of certain localized surveillance efforts rather than a total absence of these species from the entire province. This is indicated by the qualifier “in several surveys”. The occurrence of the two species in Kurdistan Province shown in Figure 2 of [2] is based on other eligible studies within our review window that recorded their presence. Thus, there is no inconsistency between the text and the map, as the text describes outcomes of specific surveys while the map aggregates all valid data across the entire review period.

Regarding the habitats of *Ae. caspius*, we agree that the original wording may have been interpreted as suggesting exceptionally high densities of this species specifically, whereas our intention was to refer to the high abundance of adult culicidae mosquitoes recorded in Qom in 2025 [9]. We also acknowledge that the term “adaptability” would benefit from further clarification. By using this term, we did not intend to imply a preference for arid or semi-arid environments, but rather the ability of *Ae. caspius* to tolerate and persist under such conditions. This interpretation is supported by previous reports showing that *Ae. caspius* has a broad ecological range across the Palearctic region and can occur in diverse aquatic habitats, including freshwater and low-salinity marshes [10]. Its use of larval habitats ranging from freshwater to brackish waters [11] further supports this interpretation.

## Concluding Remarks

We appreciate the commentator’s careful evaluation of our review and the opportunity to clarify several points. We acknowledge that a small number of relevant records were inadvertently omitted and that these omissions affected the representation of certain provinces in Figure 2 of [2]. At the same time, we maintain that the overall patterns and conclusions of the review remain supported by the available evidence and are not materially altered by these corrections. Overall, we are grateful for the constructive comments and for the opportunity to further improve the interpretation and presentation of the available evidence on the distribution of *Aedes* mosquitoes in Iran.

## Data Availability

Data sharing is not applicable to this article as no new datasets were generated or analyzed in this study.

## References

[1] Asgarian T.S., Sedaghat M.M. (2026). Comment on Nejati et al. Spatiotemporal Patterns and Historical Overview of *Aedes* Mosquitoes in Iran: A Systematic Review. *Trop. Med. Infect. Dis.* 2026, *11*, 131. Trop. Med. Infect. Dis..

[2] Nejati J., Saghafipour A., Sarvi M., Bueno-Marí R. (2026). Spatiotemporal Patterns and Historical Overview of *Aedes* Mosquitoes in Iran: A Systematic Review. Trop. Med. Infect. Dis..

[3] Harbach R.E., Wilkerson R.C. (2023). The insupportable validity of mosquito subspecies (Diptera: Culicidae) and their exclusion from culicid classification. Zootaxa.

[4] Azari-Hamidian S., Harbach R.E. (2025). Updated checklist of the mosquitoes (Diptera: Culicidae) known to occur in Iran, with updated keys to the genera, subgenera and species of *Aedes*. Zootaxa.

[5] Monchadskii A.S. (1951). The larvae of bloodsucking mosquitoes of the USSR and adjoining countries (Subfm. Culicinae). Opred. Faune SSR Moscow..

[6] Keshavarzi D., Soltani Z., Ebrahimi M., Soltani A., Nutifafa G.G., Soltani F., Faramarzi H., Amraee K., Hassanzadeh A. (2017). Monthly prevalence and diversity of mosquitoes (Diptera: Culicidae) in Fars Province, southern Iran. Asian. Pac. J. Trop. Dis..

[7] Kayedi M.H., Sepahvand F., Mostafavi E., Chinikar S., Mokhayeri H., Chegheni Sharafi A., Wong G., Shahhosseini N., Kazemi S.H. (2020). Morphological and molecular identification of Culicidae mosquitoes (Diptera: Culicidae) in Lorestan Province, Western Iran. Heliyon.

[8] Gutsevich A.V. (1943). On the mosquitoes of north Iran. Dokl. Akad. Nauk SSSR.

[9] Abedi-Astaneh F., Rad H.R., Izanlou H., Hosseinalipour S.A., Hamta A., Eshaghieh M., Ebrahimi M., Ansari-Cheshmeh M.A., Pouriayevali M.H., Salehi-Vaziri M. (2025). Extensive surveillance of mosquitoes and molecular investigation of arboviruses in Central Iran. Ann. Med. Surg..

[10] Paksa A., Sedaghat M.M., Vatandoost H., Yaghoobi-Ershadi M.R., Moosa-Kazemi S.H., Hazratian T., Sanei-Dehkordi A., Oshaghi M.A. (2019). Biodiversity of Mosquitoes (Diptera: Culicidae) with Emphasis on Potential Arbovirus Vectors in East Azerbaijan Province, Northwestern Iran. J. Arthropod-Borne Dis..

[11] Zaim M. (1987). The distribution and larval habitat characteristics of Iranian Culicinae. J. Am. Mosq. Control Assoc..

